# Solving difficulties in transseptal sheath crossing: The *shoehorn* technique

**DOI:** 10.1002/joa3.12943

**Published:** 2023-10-19

**Authors:** Juan Benezet‐Mazuecos, Álvaro Lozano, Julián Crosa, Ángel Miracle

**Affiliations:** ^1^ Arrhythmia Unit, Department of Cardiology Hospital Universitario La Luz Madrid Spain

**Keywords:** atrial fibrillation, cryoablation, transseptal puncture

## Abstract

The *shoehorn* technique is a simple and safe maneuver that can help to solve difficulties in challenging transseptal sheath crossing for atrial fibrillation cryoablation procedures.

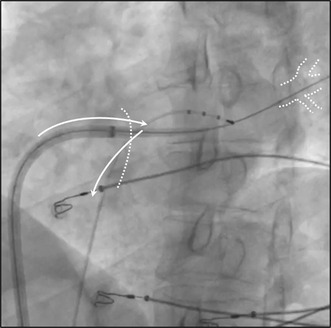

A 55 year‐old male patient diagnosed with symptomatic persistent atrial fibrillation with clinical recurrences during treatment with amiodarone was admitted for scheduled pulmonary vein (PV) isolation using cryoablation. Prior transthoracic echocardiogram showed normal left ventricle systolic function and moderate left atrium (LA) enlargement. CT‐scan showed a normal PV anatomy, and transesophageal echocardiogram showed the absence of thrombus in the LA appendage and no apparent patent foramen ovale. Cryoablation procedure was initiated using the conventional approach. Transseptal puncture was performed with a SL0 sheath and a BRK‐1 needle under fluoroscopy, guided by a wire in the aorta and a tetrapolar catheter in the coronary sinus as landmarks, and using contrast to show the tenting on the fossa ovalis before the puncture. Once it was performed, a guide wire was positioned in the left superior PV. The SL0 sheath was exchanged and the FlexCath Advance™ Steerable Sheath (Medtronic, MN, USA) was progressed over the wire. When we tried to progress the FlexCath into the LA through the wire, we found a high resistance at the atrial septum. Despite several attempts pushing with different torques, the force was insufficiently transmitted and we could not get into de LA with the sheath until we performed the *shoehorn* maneuver (Figure 1). Once this problem was solved, the procedure continued successfully without other incidents.

**FIGURE 1:**
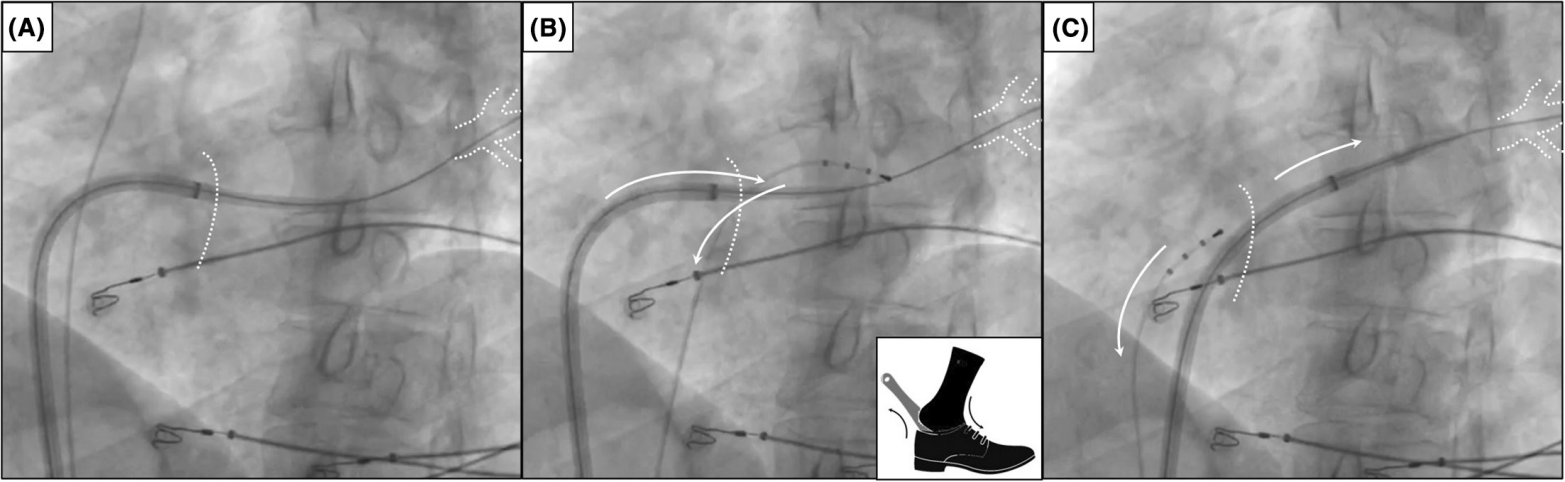
The s*hoehorn* technique. (A) Left anterior oblique fluoroscopic projection showing a tetrapolar electrode catheter placed in the right subclavian vein for phrenic nerve stimulation and the 12F FlexCath Advance^TM^ Steerable Sheath showing difficulties for transseptal crossing over a wire placed in the left superior PV. (B) The tetrapolar electrode catheter is introduced then into the LA through the transseptal puncture hole. The s*hoehorn* technique consists of removing back the tetrapolar electrode catheter while we try to progress forward the sheath over the wire placed in the left superior PV. (C) The sheath cross safely the transseptal puncture once the tetrapolar electrode is removed back into the right atrium. LA, left atrium; PV, pulmonary veins.

In this article, we present the *shoehorn* technique to solve difficulties in transseptal sheath crossing in cryoablation procedures. First, we place the tetrapolar electrode, commonly used in cryoablation procedures for right phrenic nerve pacing during right PVs ablation, in the left atrium through the transseptal puncture hole. The *shoehorn* maneuver consists in pulling back this tetrapolar electrode while, at the same time, we press and try to progress the FlexCath sheath into the transseptal hole through the wire placed in the left PV. This simultaneous and in opposing‐direction movement of the catheters at the transseptal hole expands it or minimally tears it permitting the FlexCath sheath to cross safely the transseptal puncture once the tetrapolar catheter is removed to the right atrium. Sometimes this movement should be performed more than once, especially in cases with elastic septum. In our experience, this is an easy and safe maneuver that we have successfully used in seven cases so far, with no complication and using the standard tools for the procedure (Video S1).

Transseptal puncture approach is increasing because of new and complex procedures to treat different cardiac conditions.^1^ It is a demanding procedural step in accessing the LA with inherent risks and safety concerns. Transseptal sheath crossing sometimes can be challenging. The s*hoehorn* technique is a simple maneuver successfully used to solve difficulties in transseptal access, not previously reported in cryoablation procedures.^2^

Transseptal puncture is commonly performed with fluoroscopic guidance, contrast injection, and pressure monitoring. There are many techniques for transseptal puncture facilitating difficult cases and/or allowing a more accurate position in the fossa ovalis according to the procedure (intracardiac echocardiogram, transoesophageal echocardiography, radiofrequency needle for transseptal puncture, steerable transseptal catheter with a localization/stabilization tool to identify and then navigate on the fossa…) On the contrary, these tools are not widely available over the world and add important costs to the procedure. Therefore, despite refinements of conventional instruments and innovations, traditional tools remain effective in creating and dilating the initial puncture, with an acceptable safety profile.^3^ But even for skilled operators, transseptal sheath crossing can sometimes be challenging, especially in muscular, fibrotic, or too elastic interatrial septum.^4^ Moreover, the sheath used is a 12F sheath as the FlexCath Advance™ for pulmonary vein isolation using cryoablation technology. This sheath has a slightly abrupt transition between the sheath and the dilator with a little step that sometimes causes difficulties in crossing the transseptal hole. Frequently, we experience cases where it is difficult to advance it across the septum, because of insufficient support or requiring excessive force or torque that might compromise the safety of the procedure. In these cases, we have successfully and safely used the *shoehorn* technique for transseptal sheath crossing.

The *shoehorn* technique is a simple and safe maneuver that can help to solve difficulties in transseptal sheath crossing, using the conventional tools used for a standard cryoablation procedure, and therefore avoiding unnecessary assistance/tools and saving costs.

## CONFLICT OF INTEREST STATEMENT

Authors declare no conflict of interests for this article.

## INFORMED CONSENT

Informed consent of the patient was obtained to use images to illustrate the technique.

## Supporting information


Video S1.
